# Store-and-Forward Teledermatology Wound Checks Following Mohs Surgery: A Pilot Study

**DOI:** 10.1089/tmr.2024.0039

**Published:** 2024-08-13

**Authors:** Tyler Werbel, Navid Farahbakhsh, Sailesh Konda

**Affiliations:** Department of Dermatology, UF Health Dermatology-Springhill, University of Florida, Gainesville, Florida, USA.

**Keywords:** store-and-forward, teledermatology, Mohs micrographic surgery, wound check, postoperative

## Abstract

**Introduction:**

Store-and-forward telemedicine is a form of electronic transmission in which patient images or clinical information are transmitted to clinicians for asynchronous clinical decision making. This study evaluated the use, feasibility, savings, and patient satisfaction of postoperative store-and-forward wound checks following Mohs surgery.

**Methods:**

Select patients were asked to participate in a virtual postoperative wound check after receiving Mohs surgery. Participants sent photos of their wound site and subsequently completed an anonymous survey.

**Results:**

One hundred and ten patients were enrolled, mean age 68 (range 32–87). Patients saved an average of $14.16, 78.6 miles of travel, and 90 min of travel time. Ninety-eight percent of respondents felt their quality of care in teledermatology was “about the same” to “much better” than compared with traditional in-person care. Sixty-four of the respondents (90.1%) reported they would prefer their next Mohs wound check to be conducted through teledermatology.

**Conclusion:**

Store-and-forward telemedicine in patients undergoing Mohs micrographic surgery is a safe, effective, and efficient method for postoperative wound care.

## Introduction

In 2020, there was a 63-fold increase in the number of Medicare telehealth visits (840,000 in 2019 to nearly 52.7 million in 2020).^1^ These visits have allowed patients increased access to physicians and flexibility with scheduling, while reducing wait times and travel costs. There are numerous forms of telehealth services, including video conferencing, telephone communication, e-mail or text messages, and store-and-forward telemedicine/imaging. In dermatology, there are two primary methods of telemedicine: real-time (synchronous) and store-and-forward (asynchronous) telemedicine (SFT).^2^

Store-and-forward telemedicine is a form of electronic transmission in which patient images or clinical information are transmitted to clinicians for asynchronous clinical decision making. The integration of SFT has increased in recent years and has been successfully used in the field of dermatology. Many previous studies have shown that SFT improves patient care and reduces unwarranted office visits.^3–9^ A systematic review found that teledermatology reduces wait times, allows for earlier assessment and treatment, and receives high patient satisfaction.^10^ Physicians also benefit from SFT. One study found teledermatology consultations allowed general practitioners to reduce subsequent referrals to dermatologists by approximately 20%.^4^

In practice, it offers the benefits of increased flexibility, lower cost, shorter wait times, more accessible care, and improved privacy and comfort.^1^ Nelson et al.^3^ conducted a prospective study on teledermatology in underserved settings. Their findings revealed that 77% of consultations sent from underserved primary clinics to an academic dermatology practice were effectively managed through teledermatology alone. Additionally, 61% of these consultations would not have received dermatological input otherwise.^3^ SFT has also been used for similar reasons for postoperative care in rural areas with decreased access to medical resources.^1^ While numerous studies have highlighted the benefits of SFT in general dermatology, there has been limited research on its utility and advantages in Mohs micrographic surgery (MMS), which is the gold standard for treating nonmelanoma skin cancers. MMS is a promising candidate for SFT during the postoperative period. First, the complication rate for MMS is estimated to be less than 1% and therefore rarely requires intervention postoperatively.^11^ Second, MMS is an outpatient surgical procedure that does not require postoperative hospitalization, a predictor for SFT-amenable care.^2^ Thus, this study aims to evaluate the use, feasibility, and patient satisfaction of postoperative SFT wound checks following MMS. In addition, overall travel savings were calculated based on time and mileage.

## Materials and Methods

This study was approved by the University of Florida Institutional Review Board. Study enrollment occurred between August 2022 and January 2024 at the University of Florida Department of Dermatology. Patients were asked to participate if they were ≥18 years of age, English speaking, and treated with MMS that was followed by (1) second intent healing, (2) reconstruction with absorbable sutures, or (3) reconstruction with nonabsorbable sutures and the patient can remove sutures at home. Photographically sensitive locations, such as the genitalia, and difficult-to-access surgical sites, such as the back, were excluded. Patients without internet access, an email address, or a smartphone with a camera were excluded. Demographic data, Mohs case characteristics, and comorbidities were obtained by chart review. A cost and time-saving analysis was performed using the patient’s home address, Google Maps, and the January 2022 Internal Revenue Service (IRS) standard medical mileage rate ($0.18 per mile).

**FIG. 1.**
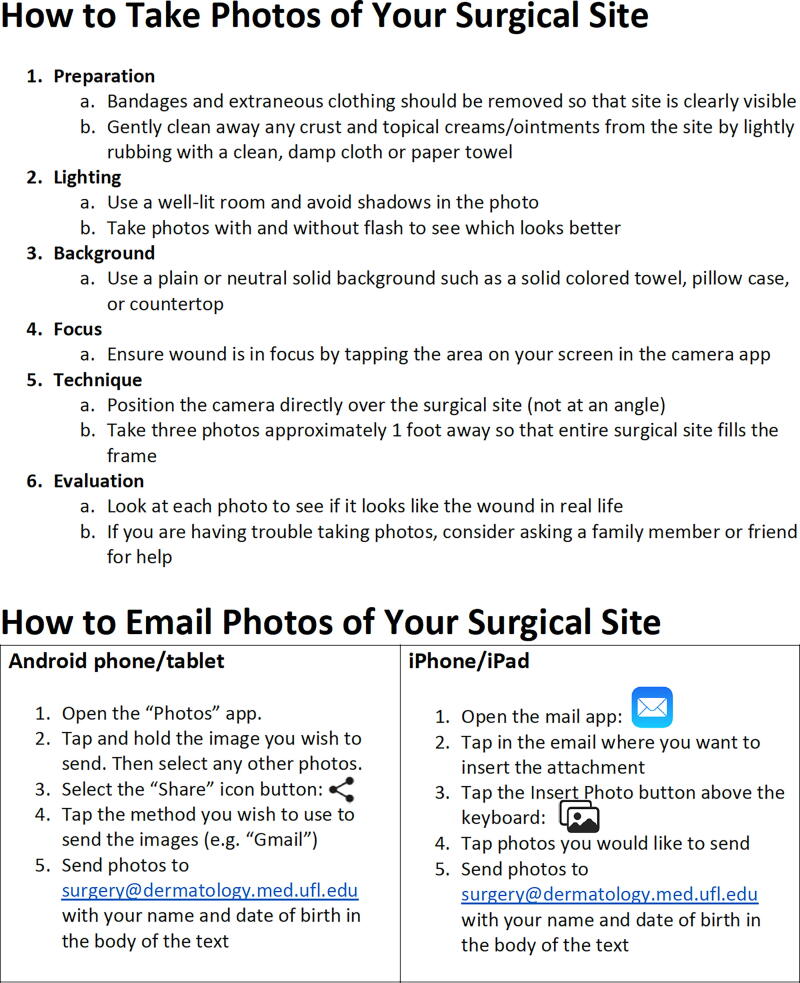
Patient handout

Patients who consented to the study received a handout (Fig. 1) following surgery that detailed how to take high-quality photographs with their personal phones. During recruitment, the attending physician or Mohs fellow used the patient handout to instruct the participant on taking high-quality photographs. If any concerns or questions arose during this teaching period, the attending physician or Mohs fellow used the patient’s smartphone to demonstrate. To emulate our institution’s in-person wound checks, patients who received sutures were contacted one week after MMS, and those who healed by second intent were contacted three and six weeks after MMS. For each virtual wound check encounter, an encrypted email was sent to the patient requesting them to respond with three photographs of their surgical site. Once the photographs were received, they were assessed for adequate quality by the Mohs fellow, and additional photos were requested if necessary. Within 72 hours of receipt, the photographs were reviewed by the attending surgeon, who provided management recommendations and assessed the need for an in-person evaluation. One to two weeks following the last store-and-forward wound check encounter, a 19-question anonymous survey was emailed to the participants that assessed their experience (Supplemental Data S1).

## Results

Our study enrolled 110 patients, mean age 68 (range 32–87), with 117 unique sites. Thirteen patients did not provide postoperative photographs and were excluded from the study. Ninety-seven patients completed the study, with 103 unique surgical sites. A variety of surgical sites were included in the study such as the face, scalp, ears, neck, and hands. Patient demographics and surgical characteristics are listed in Table 1. The average cost savings, directly related to travel, in our study was $14.16 per patient (standard deviation $17.20 and range $0.74 - $122.58). Patients also saved an average of 78.6 miles (standard deviation 95.6 miles and range 4.1 - 681 miles) and 90 min of travel time by participating in this study. The most common benefit (35.8%) reported in our study was decreased travel time.

**Table 1.** Patient Demographic and Surgical Characteristics

Patient demographics		
Sex	Count	Percentage
Male	62	60.2%
Female	41	39.8%

Avg. age	68
Median	71.0
Range	32 to 87
Avg. stages	1.29
Avg. preop size (cm)	0.93 × 1.00
Avg. postop size (cm)	1.39 × 1.54

	

Closure	Count
Closure	Count
Linear (average suture length 4.95 cm)	66
Secondary intention	25
Transposition	6
Purse	3
Advancement	3
Total	103

Twelve participants (12.3%) experienced a postoperative complication. Mild dehiscence was the most common complication (33%); other minor complications included spitting sutures, delayed healing, and hypergranulation. Of the 12 complications, three patients were smokers, one was immunocompromised, and two were on antiplatelet medication.

After completing the study, participants were sent an anonymous survey to assess their experience with SFT. Seventy-five participants completed this survey. Ninety-seven percent of respondents were “very satisfied” or “satisfied” with their overall teledermatology experience. Sixty-three of the surveyed participants (84%) had never previously sent a photo to a physician. While this was most participants’ first time using this type of technology, only nine participants (12.5%) reported experiencing any technical problems. The most commonly reported problem was difficulty with emailing the photographs to the physician.

More than 95% of respondents had no privacy concerns related to their health care data. Ninety-eight percent of respondents felt their quality of care in teledermatology was “about the same” to “much better” than compared with traditional in-person care. Sixty-four of the respondents (90.1%) reported they would prefer their next Mohs wound check to be conducted through teledermatology. Survey responses can be seen in Table 2.

**Table 2.** Postsurvey Results

Survey question	Survey answers	Count (%)
Age	Average 67; Std. deviation 10.8	
Gender	Male	42
	Female	33
Education level	Less than high school degree	2 (2.7)
	High school degree	20 (26.7)
	Vocational school or associate degree	15 (20)
	Undergraduate degree	17 (22.7)
	Graduate degree	21 (28)
Was this the first Mohs surgery that you have had?	Yes	24 (32)
	No	51 (68)
Which device did you use for this study? (Check all that apply)	Apple iPhone	43 (53.1)
	Apple Mac (laptop or desktop)	2 (2.5)
	Apple iPad	3 (3.7)
	Windows desktop	6 (7.4)
	Android smart phone	23 (28.4)
	Windows tablet	0
	Other:	4 (4.9)
Anatomical location	Area H^*^	37 (35.9)
	Area M^**^	64 (62.1)
	Area L^***^	2 (1.9)
Was this the first time that you have sent photos to a physician to review as a part of your health care?	Yes	63 (84)
	No	12 (16)
How satisfied were you with the overall teledermatology experience?	Very Satisfied	54 (75)
	Satisfied	16 (22.2)
	Neutral	1 (1.4)
	Dissatisfied	0
	Very dissatisfied	1 (1.4)
How comfortable did you feel with your teledermatology experience?	Very comfortable	63 (87.5)
	Somewhat comfortable	9 (12.5)
	Neutral	0
	Somewhat uncomfortable	0
	Very uncomfortable	0
How would you describe the quality of care in your teledermatology experience compared with traditional in-person care?	Much better	11 (15.3)
	Better	12 (16.7)
	About the same	48 (66.7)
	Worse	1 (1.4)
	Much worse	0
How would you describe the convenience of your teledermatology experience compared with traditional in-person care?	Much better	40 (55.6)
	Better	25 (34.7)
	About the same	7 (9.7)
	Worse	0
	Much worse	0
What were the greatest benefits of your teledermatology experience? (Pick all that apply)	Decreased travel time	64 (35.8)
	Decreased costs associated with travel	45 (25.1)
	Flexibility of schedule	52 (29.1)
	Avoiding missed work or school	18 (10.1)
Did you experience any technical problems or challenges during your teledermatology experience?	Yes	9 (12.5)
	No	63 (87.5)
What technical problems or challenges did you experience? Pick all that apply.	Difficulty taking adequate photographs	2 (15.4)
	Poor camera	1 (7.7)
	Emailing photographs to the physician	6 (46.2)
	Poor WiFi Connection	1 (7.7)
	Slow device	1 (7.7)
	Other:	2 (15.4)
I was concerned about the security of sending personal health information by email.	Strongly disagree	38 (53.5)
	disagree	16 (22.5)
	Neither agree or disagree	15 (21.1)
	Agree	2 (2.8)
	Strongly agree	0
I was concerned about the privacy of my health care data.	Strongly disagree	37 (52.1)
	disagree	15 (21.1)
	Neither agree or disagree	17 (23.9)
	Agree	2 (2.8)
	Strongly agree	0
I was concerned about how my personal health data would be used.	Strongly disagree	37 (52.1)
	disagree	16 (22.5)
	Neither agree or disagree	15 (21.1)
	Agree	3 (4.23)
	Strongly agree	0
Would you prefer your next Mohs wound check to be conducted in-person or by teledermatology?	Teledermatology	64 (90.1)
	In-person	7 (9.9)

^*^ central face, eyelids, eyebrows, nose, lips, chin, ear, preauricular, genitalia, hands, feet.

^**^ cheeks, forehead, scalp, neck, jawline, pretibial surface.

^***^ trunk, extremities.

## Discussion

The use of telehealth medicine, including synchronous and asynchronous methods (such as store-and-forward) has significantly increased in the field of dermatology. SFT has been shown to enhance patient care through convenience, accessibility, patient satisfaction, and unnecessary office visits.^3–10^ Before the COVID-19 pandemic, only 23% of Mohs surgeons were utilizing telemedicine.^12^ This number rose to 86% during the pandemic as Mohs surgeons began to utilize telemedicine primarily for postsurgical management and surgical consultation. Our study highlights that the use of SFT for postoperative wound care in MMS is noninferior to in-person visits and oftentimes patients prefer this method of postoperative care. SFT can lead to improved patient access, care, and satisfaction.^13–16^ Previous studies on MMS have looked at the role of telemedicine in preoperative consultations and more recently postoperative wound care.^14,17^ This is the first study that evaluates specific patient demographics, risk factors, complications, distance traveled, time and cost saved, and specific characteristics of their Mohs surgery in addition to postoperative surveys related to SFT in MMS postoperative wound care.

The main benefit for patients in SFT is cost savings and reduced travel time. A study on preoperative consultation before MMS found that patients who used SFT saved 163 min and 145 miles of travel time, and approximately $60.00 per person.^17^ While our study had a lower average cost of savings ($14.16) it did not take into account other various areas of financial loss, including money lost from missing work, wear and tear on personal transportation, hiring childcare to attend an appointment, or the cost of food during long commutes.

The amount of time participants saved using SFT is also conservative, as it does not include parking, checking-in, and the office visit itself, which can take an additional 15–30 min. For this reason, it can be assumed that patients were able to save substantially more time by participating in virtual postoperative care. This is similar to previous postoperative surgical studies, which have found online visits taking significantly less time for patients than in-office visits (15 vs. 103 min).^15^

Travel time can be a major burden and barrier for patients receiving care. Reduced travel time is one of the major benefits of SFT and patients made this a point of emphasis on the postsurvey. Certain patient populations may be disproportionately affected by travel distance. Beltrami et al. reported that nearly 15% of Medicaid beneficiaries have a travel time ≥ 60 min compared with 8.4% of Medicare beneficiaries.^18^ The savings in cost and time should not be overlooked as SFT may be a way to improve patient access, satisfaction, and compliance.

Patient satisfaction and overall experience are crucial to the successful implementation of SFT.^10^ Numerous factors contribute to patient satisfaction. Chang et al. noted disparities in satisfaction among older and non-white dermatology patients.^19^ Their study reported that older patients (age >= 66) were more dissatisfied with telemedicine and that non-white race was associated with greater concerns for privacy, and inappropriate access to medical information.^19^ On the contrary, our study found that older patients were very satisfied with telemedicine and would be willing to try it again in the future. While these factors should be taken into consideration when scheduling a postoperative wound check, our study determined that the majority of patients believe their quality of care in teledermatology was the same, if not better, when compared with traditional in-person visits. Also, after completing one SFT wound check visit, patients are more likely (90%) to prefer this method in the future. This is similar to a 2024 study that reported roughly 75% of patients preferred electronic follow-up.^14^

While SFT can provide a significant benefit to patients and Mohs surgeons, it is not without its challenges. A common problem encountered by dermatologists in SFT is insufficient image quality. One teledermatology study examined 3,600 patient-submitted images and found that only 62.2% of the images were of sufficient quality and 55.1% were considered useful for decision making.^13^ In our study, the majority of respondents (84%) had never previously sent a photo to a physician for review. To reduce the number of poor-quality images taken, a postprocedure handout can be provided to patients educating them on how to take high-quality photographs. This handout subsequently reduced the number of poor-quality images, in our study, as only eight submitted photos were inadequate (8.2%). In addition, upon request, these eight patients successfully resubmitted higher quality images.

High-quality photos can be used for diagnostic purposes. One study found the specificity of photograph reviews for the diagnosis of surgical site infections to be 90%.^20^ However, in that study physicians and researchers took the postoperative photographs of the wound site, effectively eliminating the risk for poor-quality images. When implementing SFT, it is important to provide patients with instructions on how to take high-quality photographs. A 2010 study on the feasibility of SFT in dermatology found that after an oral or electronic tutorial on submitting digital images, nearly three-quarters of patient images were focused and of high quality.^21^

MMS is safe and has a low complication rate. With adequate image quality, the surgeon can adequately assess for postoperative complications. All complications in our study were minor and the most common was mild dehiscence. Five patients were offered in-person visits for evaluation, as the rest were managed with observation. Previous studies have also shown that postoperative complications can be identified through online visits and that it is not inferior to in-person exams.^15^ Surgeons can also determine which patients are at higher risk for postoperative wound infections before recommending SFT. Patient comorbidities, such as smoking status, immunosuppression, and medication history can be used to risk-stratify surgical patients, who are at higher risk for postoperative wound infections.

While SFT is safe and cost-effective, there are still limitations to its implementation. Barriers include patient adherence, image quality, scheduling workflow, staff and patient training, physical exam limitations, and physicians’ individual experience and comfort level in reviewing clinical images. A limitation in our study includes selection bias, as only participants who felt comfortable using a mobile device and those who had access to one were selected. This is similar to prior studies^14^; however, this is the first study that assesses the specific time and cost savings, as well as evaluates surgical characteristics and complications. Another limitation is that more than two-thirds of the study participants previously had Mohs surgery. This could mean that these patients were more comfortable with the procedure and expectations postoperatively.

## Conclusion

SFT in patients undergoing MMS is a safe, effective, and efficient method for postoperative wound care. There are numerous benefits for the patient, including significantly reduced travel time and cost savings. Patients are satisfied with this form of postoperative care and some prefer it over an in-person visit. Patient education is critical to ensure high-quality photographs are provided. Mohs surgeons also benefit from SFT as they can improve workflow and efficiently assess postoperative wound care and potential complications. While some patients may still prefer an in-person exam, SFT can be used as a viable alternative for select patients.

## Data Availability

The data that support the findings of this study are available from the corresponding author (S.K.) upon request.

## Abbreviations Used

IRS: Internal Revenue Service
MMS: Mohs micrographic surgery
SFT: Store-and-forward telemedicine
