# Supplementary material for: Store-and-Forward Teledermatology Wound Checks Following Mohs Surgery: A Pilot Study
Source: Telemed Rep. 2024 Aug 13;5(1):256–62. doi: 10.1089/tmr.2024.0039 (PMC11342048; doi:10.1089/tmr.2024.0039)
Supplement: Supplemental Data S1 [file tmr.2024.0039_supplemental_file_1.pdf]

Supplemental file 1. Store-and-Forward Patient Survey

Q1 The purpose of this anonymous survey is to better understand patients' experiences with teledermatology wound checks after Mohs surgery. You will be asked a series of questions on the perceived benefits and challenges of using teledermatology for postoperative wound checks.

Q2 What is your age?

	25 30 35 40 45 50 55 60 65 70 75 80 85
Age	

Q3 What is your gender?

- Male
- Female
- Non-binary / third gender

Q4 What is your highest education level?

- Less than high school degree
- High school degree
- Vocational school or associate degree
- Undergraduate degree (4 year)
- Graduate degree

Q5 Was this the first Mohs surgery that you have had?

- Yes
- No

Q6 Which device(s) did you use for this study? Check all that apply.

- Apple iPhone
- Apple Mac (laptop or desktop)
- Apple iPad
- Windows desktop
- Android smart phone
- Windows tablet
- Other: _________

Q7 Where was the location of your Mohs surgery?

- Nose
- Cheek
- Ear
- Forehead
- Temple
- Around the eyes
- Around the mouth
- Scalp
- Front of neck
- Bk of neck
- Back
- Chest
- Abdomen
- Arm
- Hand
- Leg
- Foot
- Other: ____

Q8 Was this the first time that you have sent photos to a physician to review as a part of your healthcare?

- Yes
- No

Q9 How satisfied were you with the overall teledermatology experience?

- Very Satisfied
- Satisfied
- Neutral
- Dissatisfied
- Very dissatisfied

Q10 How comfortable did you feel with your teledermatology experience?

- Very comfortable
- Somewhat comfortable
- Neutral
- Somewhat uncomfortable
- Very uncomfortable

Q11 On a scale of 1-10 (1 the easiest, 10 the hardest), how difficult was it to share photos of your surgical wound with your physician?

	1 2 3 4 5 6 6 7 8 9 10
Slide to Answer	

Q12 How would you describe the **quality of care** in your teledermatology experience compared with traditional in-person care?

- Much better
- Better
- About the same
- Worse
- Much worse

Q13 How would you describe the **convenience** of your teledermatology experience compared with traditional in-person care?

- Much better
- Better
- About the same
- Worse
- Much worse

Q14 What were the greatest benefits of your teledermatology experience? Pick all that apply.

- Decreased travel time
- Decreased costs associated with travel
- Flexibility of schedule
- Avoiding missed work or school
- None

Q15 Did you experience any technical problems or challenges during your teledermatology experience?

- Yes
- No

Display This Question:

If Did you experience any technical problems or challenges during your teledermatology experience? = Yes

Q16 What technical problems or challenges did you experience? Pick all that apply.

- None
- Difficulty taking adequate photographs
- Poor camera
- Emailing photographs to the physician
- Poor WiFi Connection
- Slow device
- Other: ____

Q17 Rate your level of agreement with the following statements about your teledermatology experience:

	Strongly disagree	Disagree	Neither disagree nor agree	Agree	Strongly agree
I was concerned about the privacy of my healthcare data.	○	○	○	○	○
I was concerned about the security of sending personal health information by email.	○	○	○	○	○
I was concerned about how my personal health data would be used.	○	○	○	○	○

Q18 Would you prefer your next Mohs wound check to be conducted in-person or by teledermatology?

- In-person
- Teledermatology

Q19 Thank you for your participation in this study. Please write any additional comments or feedback regarding your teledermatology experience in the box below:
