# Supplementary material for: Store-and-Forward Teledermatology Wound Checks Following Mohs Surgery: A Pilot Study
Source: Telemed Rep. 2024 Aug 13;5(1):256–62. doi: 10.1089/tmr.2024.0039 (PMC11342048; doi:10.1089/tmr.2024.0039)
Supplement: Supplemental Data S2 [file tmr.2024.0039_supplemental_file_2.pdf]

Supplemental file 2. Patient Handout

How to Take Photos of Your Surgical Site

1. **Preparation**
   a. Bandages and extraneous clothing should be removed so that site is clearly visible
   b. Gently clean away any crust and topical creams/ointments from the site by lightly rubbing with a clean, damp cloth or paper towel
2. **Lighting**
   a. Use a well-lit room and avoid shadows in the photo
   b. Take photos with and without flash to see which looks better
3. **Background**
   a. Use a plain or neutral solid background such as a solid colored towel, pillow case, or countertop
4. **Focus**
   a. Ensure wound is in focus by tapping the area on your screen in the camera app
5. **Technique**
   a. Position the camera directly over the surgical site (not at an angle)
   b. Take three photos approximately 1 foot away so that entire surgical site fills the frame
6. **Evaluation**
   a. Look at each photo to see if it looks like the wound in real life
   b. If you are having trouble taking photos, consider asking a family member or friend for help

How to Email Photos of Your Surgical Site

<table>
<tr><th>Android phone/tablet</th><th>iPhone/iPad</th></tr>
<tr><td>
1. Open the “Photos” app.
2. Tap and hold the image you wish to send. Then select any other photos.
3. Select the “Share” icon button:
4. Tap the method you wish to use to send the images (e.g. “Gmail”)
5. Send photos to surgery@dermatology.med.ufl.edu with your name and date of birth in the body of the text
</td><td>
1. Open the mail app:
2. Tap in the email where you want to insert the attachment
3. Tap the Insert Photo button above the keyboard:
4. Tap photos you would like to send
5. Send photos to surgery@dermatology.med.ufl.edu with your name and date of birth in the body of the text
</td></tr>
</table>
